# Supplementary material for: Spontaneous breaking of symmetry in overlapping cell instance segmentation using diffusion models
Source: Biol Methods Protoc. 2024 Nov 9;9(1):bpae084. doi: 10.1093/biomethods/bpae084 (PMC11631529; doi:10.1093/biomethods/bpae084)
Supplement: bpae084_Supplementary_Data [file bpae084_supplementary_data.zip › combined1.pdf]

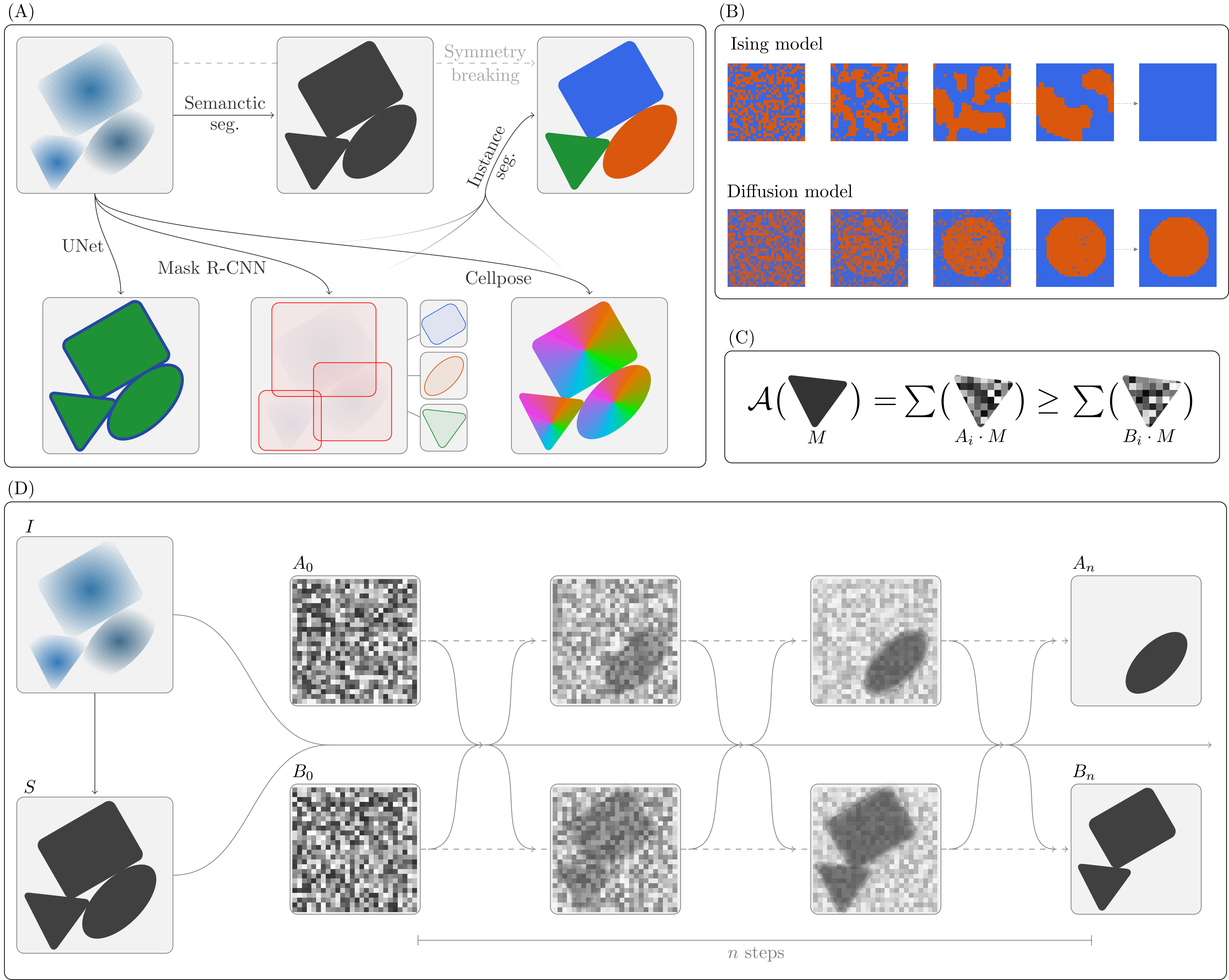
(A)
Semanctic
seg.
Symmetry
breaking
Instance
seg.
UNet
Mask R-CNN
Cellpose
(B)
Ising model
Diffusion model
(C)
$\mathcal{A}(M) = \sum(A_i \cdot M) \geq \sum(B_i \cdot M)$
(D)
I
S
A_0
A_n
B_0
B_n
n steps
