# Supplementary figures and images for: Spontaneous breaking of symmetry in overlapping cell instance segmentation using diffusion models
Source: Biol Methods Protoc. 2024 Nov 9;9(1):bpae084. doi: 10.1093/biomethods/bpae084 (PMC11631529; doi:10.1093/biomethods/bpae084)

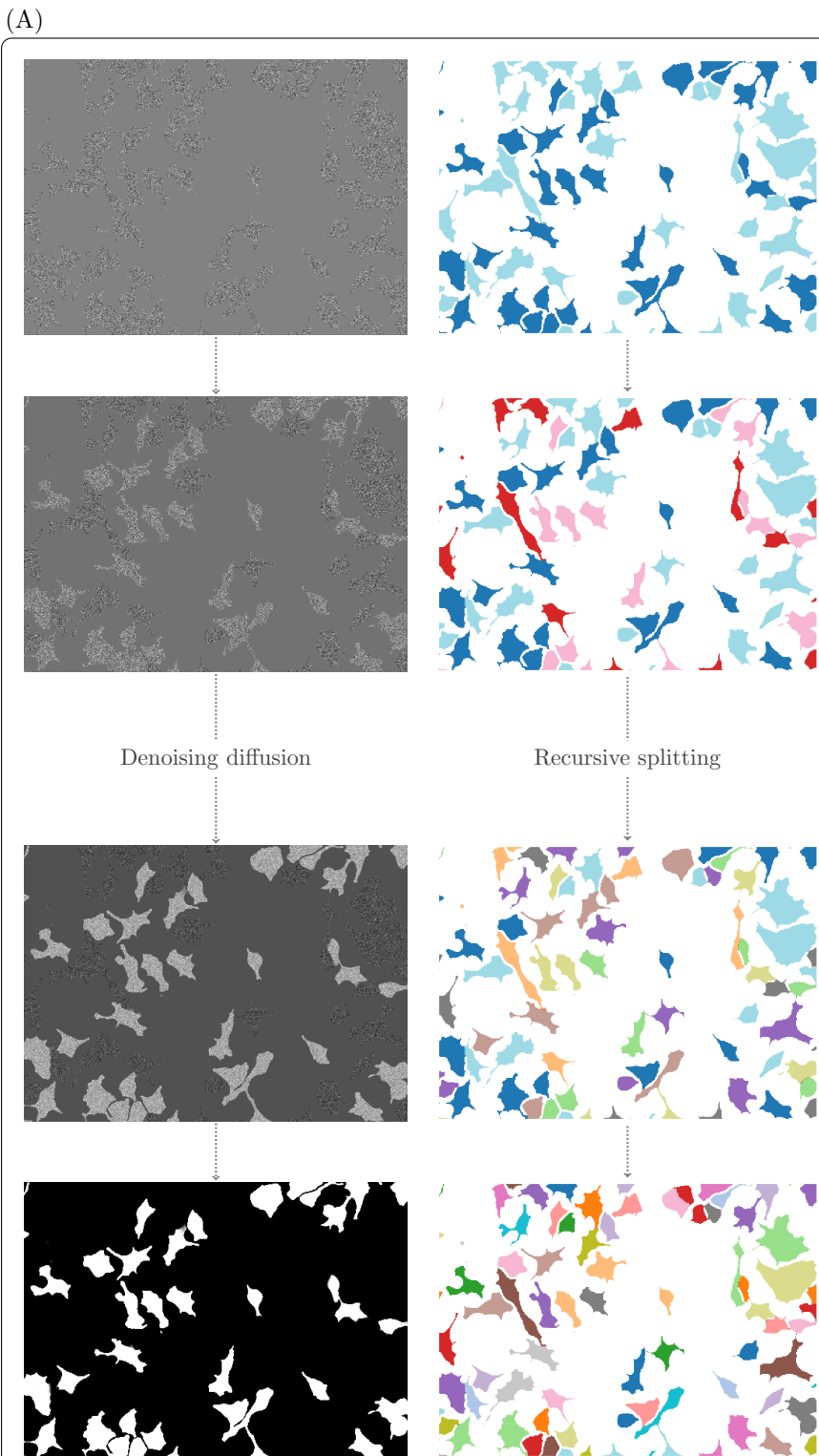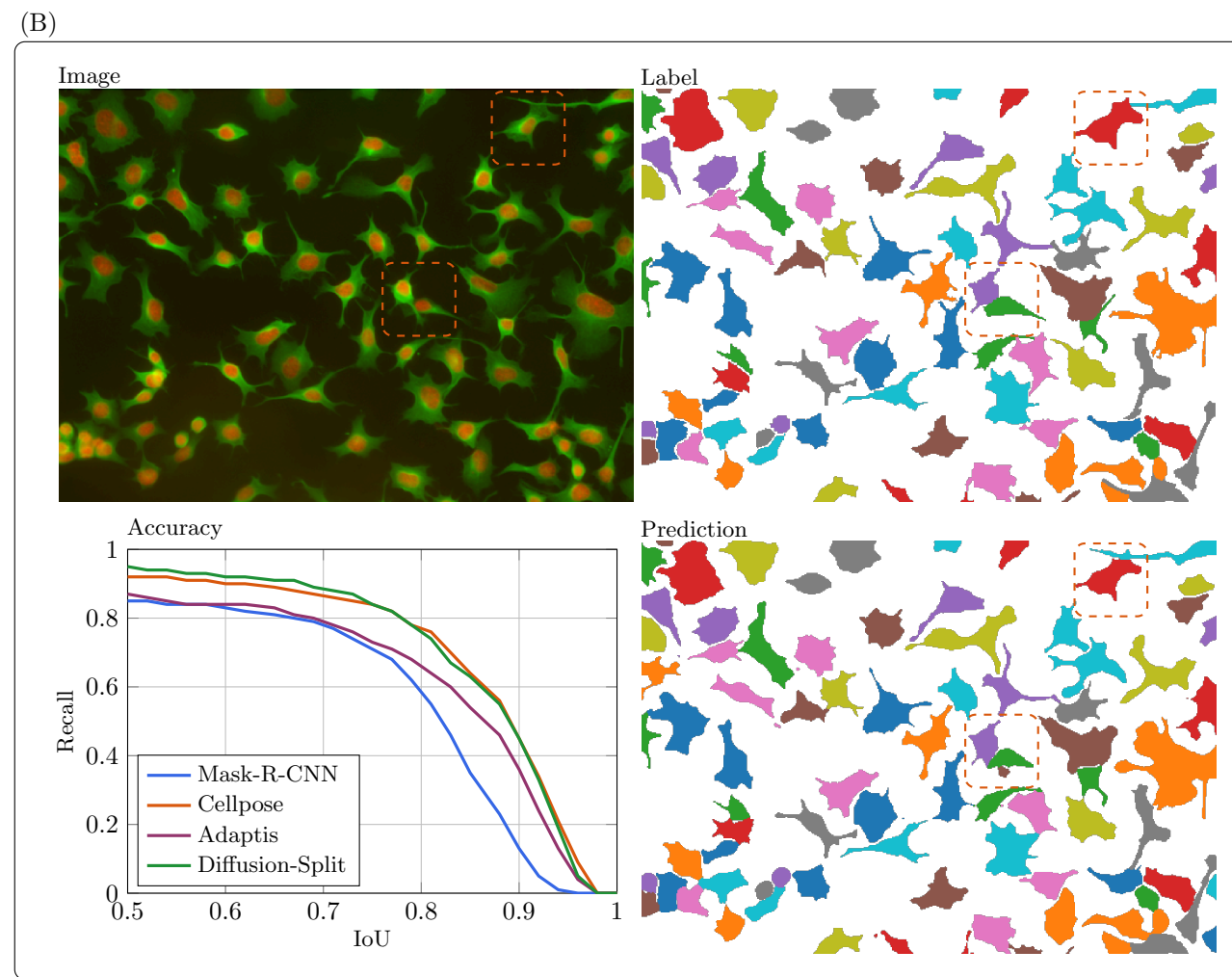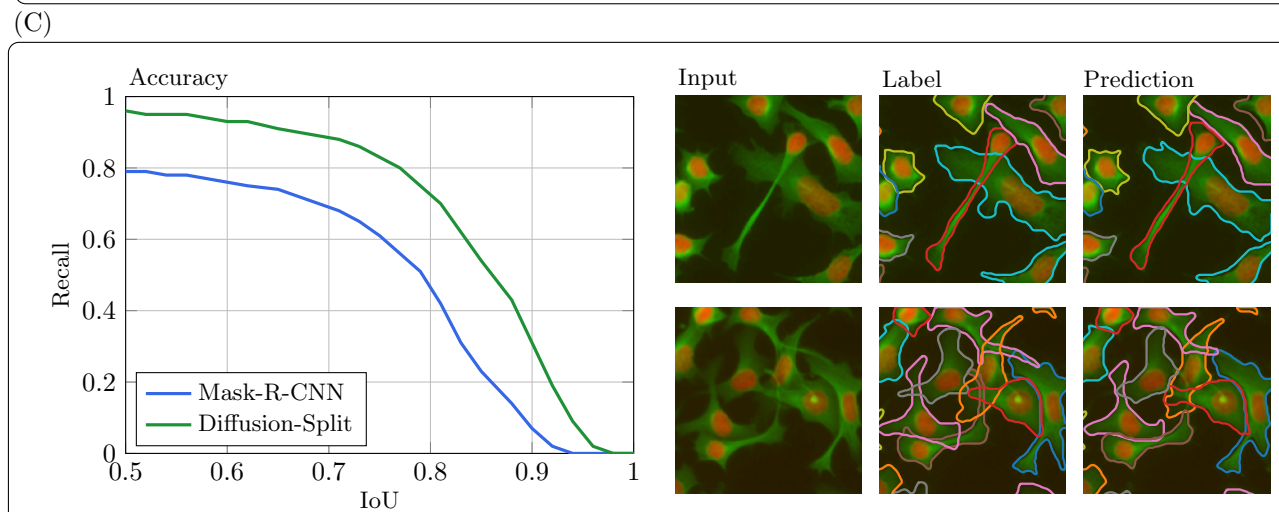

Supplement: bpae084_Supplementary_Data [file bpae084_supplementary_data.zip › combined2.pdf]
